# Fumonisin B1, B2 and B3 in Muscle and Liver of Broiler Chickens and Turkey Poults Fed with Diets Containing Fusariotoxins at the EU Maximum Tolerable Level

**DOI:** 10.3390/toxins11100590

**Published:** 2019-10-11

**Authors:** Didier Tardieu, Angelique Travel, Jean-Paul Metayer, Celeste Le Bourhis, Philippe Guerre

**Affiliations:** 1 Université de Toulouse, INP, ENVT, UR Mycotoxicologie, F-31076 Toulouse, France; d.tardieu@envt.fr; 2 ITAVI, Centre INRA Val de Loire, 37380 Nouzilly, France; travel@itavi.asso.fr; 3 ARVALIS-Institut du Végétal, Station expérimentale, 91720 Boigneville, France; M.VILARINO@arvalis.fr; 4 INRA Unité Expérimentale 1295 PEAT, Centre INRA Val de Loire, 37380 Nouzilly, France; celeste.lebourhis@inra.fr

**Keywords:** fumonisins, muscle, liver, broilers, turkeys, deoxynivalenol, zearalenone

## Abstract

Although provisional maximum tolerable daily intake and recommended guidelines have been established for fumonisins (FB) in food, few data are available concerning levels of FB in edible animal tissues. Such data are of particular interest in avian species that can tolerate relatively high levels of fumonisins in their feed. Also, even if multiple contamination of animal feed by toxins produced by *Fusarium* is very frequent, little is known about the consequences of multiple contamination for FB levels in tissues. The aim of this study was to analyze the concentrations of FB in the muscle and liver of chickens and turkeys fed with FB alone and with FB combined with deoxynivalenol (DON), and with zearalenone (ZEN). Experimental diets were formulated by incorporating ground cultured toxigenic *Fusarium* strains in corn-soybean based feeds. Control diets were free of mycotoxins, FB diets contained 20 mg FB1+FB2/kg, and FBDONZEN diets contained 20, 5, and 0.5 mg/kg of FB1+FB2, DON, and ZEN, respectively. Animals were reared in individual cages with free access to water and feed. The feed was distributed to male Ross chickens from the 1st to the 35th day of age and to male Grade Maker turkeys from the 55th to the 70th day of age. On the last day of the study, the birds were starved for eight hours, killed, and autopsied for tissues sampling. No sign of toxicity was observed. A UHPLC-MS/MS method with isotopic dilution and immunoaffinity clean-up of samples has been developed for analysis of FB in muscle (n = 8 per diet) and liver (n = 8 per diet). Only traces of FB that were below the LOQ of 0.25 µg/kg were found in most of the samples of animals fed with the control diets. Mean concentrations of FB1, FB2, and FB3 in muscle were 17.5, 3.39, and 1.26 µg/kg, respectively, in chickens, and 5.77, 1.52, and 0.54 µg/kg in turkeys, respectively. In the liver, the respective FB1, FB2, and FB3 concentrations were 44.7, 2.61, and 0.79 µg/kg in chickens, and 41.47, 4.23, and 1.41 µg/kg, in turkeys. Cumulated level of FB1+FB2+FB3 in the highly contaminated samples were above 60 and 100 µg/kg in muscle and liver, respectively. The concentrations of FB in the tissues of animals fed the FBDONZEN diet did not greatly differ from the concentrations measured in animals fed the diet containing only FB.

## 1. Introduction

Fumonisins are mycotoxins produced by Fusarium, mainly *F. verticillioides* [1,2,3]. These compounds are found worldwide, sometimes at relatively high levels in human food and animal feed [1,2]. Because of their fungal origin, not only one metabolite is produced, and 28 fumonisin analogs have been identified until now [1,2]. The most widely studied fumonisins belong to the “B” family (FB), FB1 being the most abundant, the other being FB2, FB3, and FB4 [1,2]. Other fumonisins produced by Fusarium found in food and feed are fumonisin A (FA), fumonisin C (FC), fumonisin P (FP) and hydrolyzed and partially hydrolyzed fumonisins (HFB). Although FA, FC, FP, and HFB have been shown cytotoxic and inhibit sphingolipid synthesis, FB are the most abundant and the most toxic compounds [1,2,4,5,6,7,8]. Accordingly, the provisional maximum tolerable daily intake (PMTDI) and recommended guidelines on fumonisins in food and feed have been established based on the cumulated intake of FB [1,2,5,9]. 

After their administration in animals, FB are poorly absorbed and rapidly excreted [8,10]. Only a very small amount of the administered dose is found in plasma, and the metabolism of FB appeared to be weak [8,10]. Although HFB, FA, and N-carboxymethyl FB have been found in the liver and feces of different species, the mechanism of their formation is not well understood and their contribution to the overall toxicity of FB is considered to be insignificant compared to that of the parent compound [8,11,12,13,14].

Taking into account the levels and occurrence of FB in raw materials, their poor absorption in animals and their weak level in milk, human exposure to FB through consumption of animal products and products of animal origin is considered to be negligible [1,5,8]. However, to date, no data are available on levels of FB in the muscles of poultry that can tolerate high levels of FB in their feed [2,5]. Also, as multiple contamination by toxins produced by *Fusarium* is common in poultry diets, and some fusariotoxins are known to change xenobiotic and nutriment absorption, concomitant exposure to several toxins could change the level of FB in tissues [10,15]. Specifically, deoxynivalenol (DON) is known to affect the intestinal barrier function in several animal species, which could modify the bioavailability of xenobiotics [16,17,18,19,20,21,22]. However, chronic exposure to DON appeared to have no influence on the oral bioavailability of a single dose of FB1 [23]. Concerning zearalenone (ZEN), a study in broiler chickens showed that dietary ZEN improved nutrient digestibility, suggesting FB bioavailability could change during concomitant exposure to FB and ZEN [24]. 

Several methods of analysis of fumonisins have been developed in plant and biological samples, however the UHPLC-MS/MS methods have been shown to have the highest sensitivity and specificity [25]. The objectives of these methods varies with the samples analyzed. Indeed, whereas the main objective of UHPLC-MS/MS analysis of food and feed is usually to detect multiple mycotoxins, the main objective of analysis using biological matrices is sensitivity, especially for FB, whose level in sample is generally. Most UHPLC-MS/MS methods used for the analysis of biological samples involve precipitation of proteins with organics solvents, liquid–liquid extraction and solid-phase extraction before LC-MS analysis [26,27,28,29,30]. The columns usually used to purify the samples are of the SAX or C18 type, whereas immunoaffinity (IA) columns are rarely used in UHPLC-MS/MS analysis, except to clean some plant samples [31,32]. IA columns have been used with HPLC analysis and fluorescent detection for quantitation of FB1 in liver, but the relatively low sensitivity of fluorescence detection did not enable quantitation of other FB than FB1, nor quantitation of FB1 in muscles [33].

So, although a PTDI for FB1, FB2 and FB3 of 2 µg/kg body weight has been established, no data are available on the level of FB in muscle of avian species. Because broilers and turkeys are major sources of meat and because these species can tolerate high levels of FB in feed, assessment is of special interest. Also, because of the multiple occurrence of fusariotoxins in feed, understanding the consequences of multiple exposure on the level of FB in tissues is also of interest. To this end, we first developed an UPLC-MS/MS method that enables simultaneous quantitation of FB1, FB2, and FB3 in muscle and liver. We then applied the method to analyze samples that had been taken as a part of two toxicological studies in chickens and turkey fed with FB alone, and fed with FB in combination with DON and ZEN [34,35].

## 2. Results and Discussion

### 2.1. Detection of FB and Treatment of Samples

**Figure 1:**
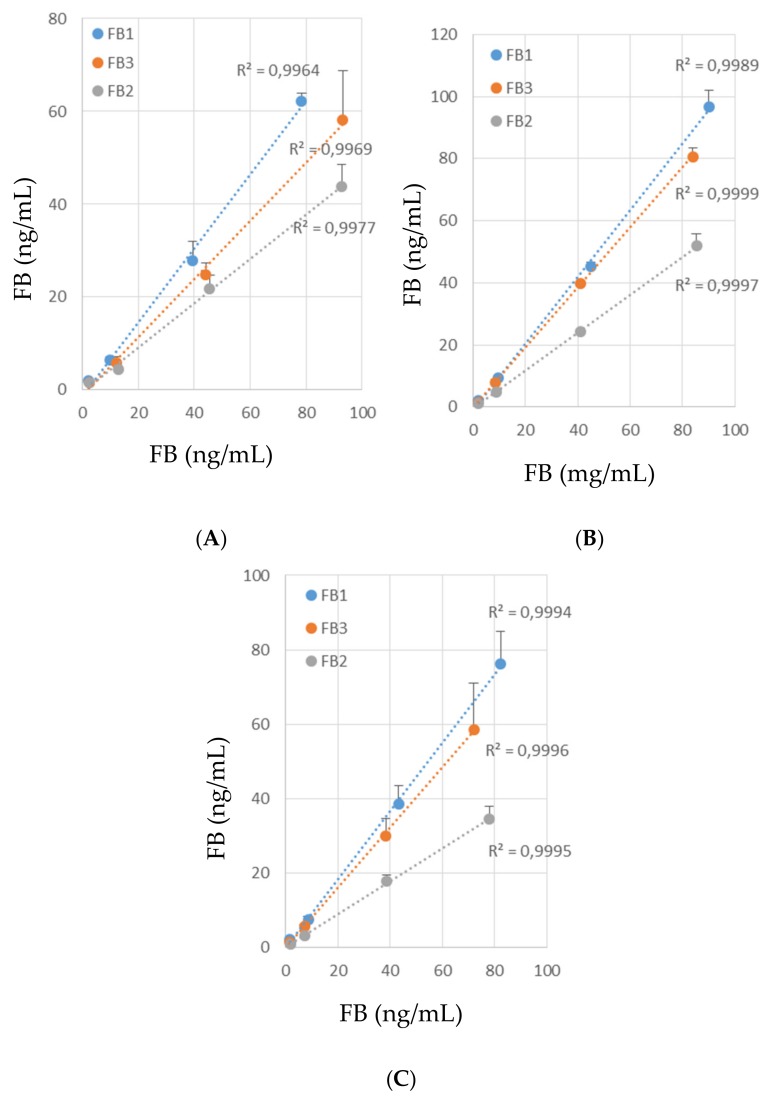
FB1, FB2, and FB3 standards recovery after immuno-affinity clean-up (**A**) and matrix effect observed after IA cleanup of muscles (**B**) and liver (**C**) obtained in chicken and turkey not exposed to FBs. Results are the mean ± SD of three replicates at four concentrations levels.

Several methods are available for the analysis of FB by LC-MS/MS, and there is a wide consensus on the detection of the analytes by positive electrospray ionization [2,14,25]. For each precursor ion, the most abundant product ion is monitored and used for quantitation (Table 1). To avoid false interpretation of results, we used not only one but two different ions for the identification of FB1, FB2, and FB3. We also used isotope dilution mass spectrometry because it is the technique that give the most precise results. Because of the rates of recovery observed in a preliminary assay after pure solutions of the analytes to measure had been passed through the IA column, isotopic dilution appeared especially necessary in this study (Figure 1A and Table 2). Indeed, while the observed rates of recovery did not differ with the concentration of FB1, FB2, and FB3 used (Figure 1A), they did differ with the toxin. Mean recovery rates were 75% and 49%, of FB1 and FB2, respectively, and the difference was statistically different (Table 2). The mean recovery rate of FB3 was 62%. The mean recovery rate measured with C13FB1 was 78%, which was not statically different from the one measured for FB1. The mean recovery rate of C13FB2 was 50%, which was not statistically different from FB2, but did differ from C13FB1. Because a recovery rate of FB1+FB2 above 70% was expected, the column was washed again. Only traces of FB were detected, less than 5% of the amount put on the column. As the recovery rate varied with the toxin assayed, but the amount of toxin put on the column did not, the low recovery rate observed with FB2 was probably due to differences in binding specificity of the column for FB1 and FB2. This result is in agreement with previous studies in which different recovery rates of FB1 and FB2 were reported for samples after the use of IA columns [31,32,36,37,38]. The use of isotope-labelled internal standards made it possible to compensate for these differences. 

In order to estimate the matrix effect, blank extracts of liver and blank extracts of muscle were prepared from chickens and turkeys fed with a diet containing less than 50 µg FB1+FB2/kg over a period of 14 days or more. In all the blank extracts, no FB was measured with appropriate abundance of the two qualifiers shown in Table 1. Matrix effects were measured by spiking the blank extracts with solutions of standards containing FB1, FB2, and FB3, at four different concentrations, and C13FB1 and C13FB2 at one concentration. Because no significant difference between species was observed, data in Figure 1B,C and Table 2 are reported as the mean ± SD observed in four chicken and four turkey samples. Matrix interactions did not differ with the concentration of FB1, FB2, and FB3 used (Figure 1B,C). The mean matrix interactions for the detection of FB1 in muscle and liver were 104 and 97%, respectively (Table 2). By contrast, the mean matrix interactions for FB2 were 59 and 46% in muscle and liver, respectively, while the mean matrix interactions for FB3 were 92 and 83%, respectively. Concerning the isotope-labelled standards, the respective mean matrix interactions in muscles and liver with C13FB1 were 98 and 95% and were 58 and 51% with C13FB2. As shown in Table 2, matrix interactions did not statistically differ with the spiked tissue but did with the toxin. Although matrix interactions with FB are not frequent, matrix suppression or matrix enhancement has already been described in some samples [14,39,40]. 

Because the recovery rates of standards after passage through the IA columns, and the matrix interactions differed between FB1 and FB2 but not between the toxin and its C13 isotope, all FB concentrations reported hereafter were calculated from the calibration curves of standard solutions of FB corrected by the specific recovery rates measured in each sample for C13FB1 and C13FB2. The concentrations in FB3 were estimated using the recovery rate measured for C13FB1 corrected by a factor of 1.3, which correspond to the ratio of the slopes observed after IA cleanup of standard solutions of FB1 and FB3 (Table 2).

### 2.2. Analysis of Muscle and Liver Spiked Samples

**Figure 2:**
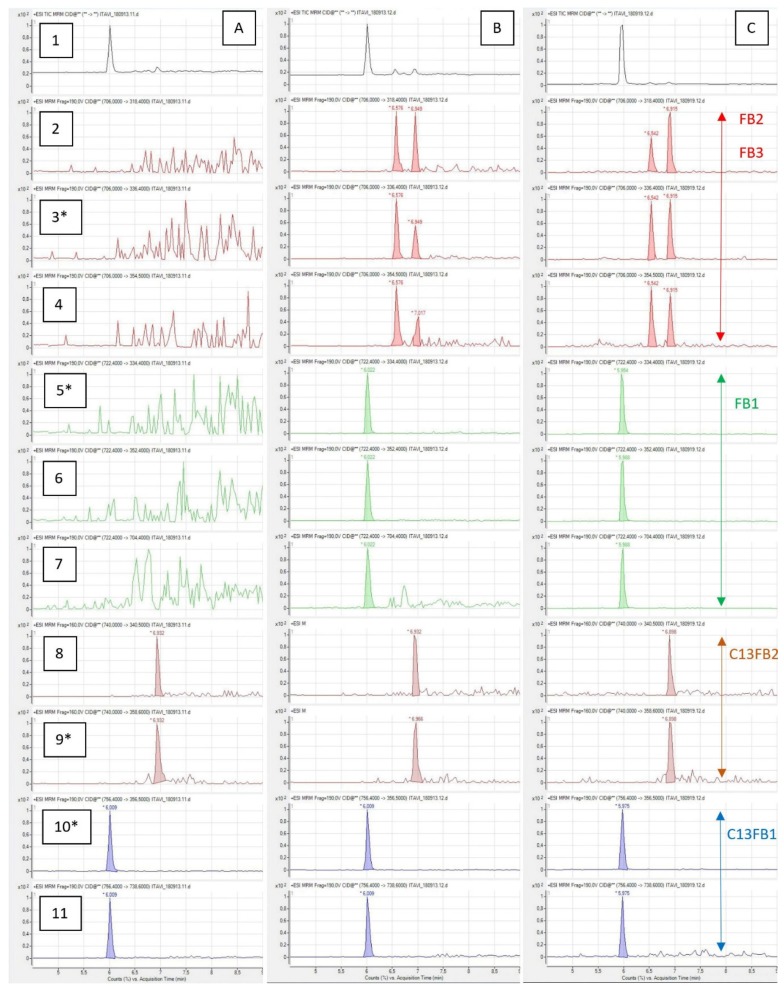
UPLC-MS/MS chromatograms of muscles spiked with 2.5µg/kg of C13FB1 and C13FB2. (**A**) blank sample; (**B**) sample spiked with FB1, FB2, and FB3 at 1 µg/kg each; (**C**) sample of turkey fed with 20 mg FB1+FB2/kg/day over a period of 14 days. Row 1: Total ions counts. Row 2: MRM 706 to 318.4; Row 3: MRM 706 to 336.4; Row 4: MRM 706 to 354.5; Row 5: MRM 722.4 to 334.4; Row 6: MRM 722.4 to 352.4; Row 7: MRM 722.4 to 704.4; Row 8: MRM 740 to 340; Row 9: MRM 740 to 358.6; Row 10: MRM 756.4 to 356.5; Row 11: MRM 756.4 to 738.6. An asterisk was used to identify the transition used to quantify the toxin.

Figure 2A shows a typical UPLC-MS/MS chromatogram of muscle spiked with 2.5 µg/kg of C13FB1 and C13FB2 each, obtained from turkey fed a diet containing less than 50 µg FB1+FB2+FB3/kg. No peak of FB1, FB2, and FB3 was found in this sample with an acceptable ratio of the ions used as qualifiers defined in Table 1, whereas C13FB1 and C13FB2 were easy to quantify. Figure 2B shows a typical chromatogram of the same muscle spiked with 1 µg/kg of FB1, FB2, and FB3 each, and 2.5 µg/kg of C13FB1 and C13FB2 each. As shown on this chromatogram, FB1, FB2, and FB3 are correctly separated and can be quantified with acceptable ratios of ± 20% of the qualifiers.

The mean recovery rates of C13FB1 in muscle and liver were 42 and 75%, respectively (Table 2). These rates of recovery are in agreement with those observed in a previous study in which IA cleanup of samples and fluorescent detection of FB1 were used. The mean percentage of extraction of FB1 in muscle and liver were 53 and 75%, respectively [33]. The mean recovery rates of C13FB2 in muscle and liver were 17 and 37% (Table 2). These results are coherent with both the lower recovery rate after IA, and the higher matrix decrease of the signal observed with C13FB2 compared with C13FB1. The rate of recovery measured for C13FB1 and C13FB2 in each sample made it possible to calculate the concentration of FB1, FB2, and FB3 (Table 2). At the concentrations assayed, the mean recovery rates of FB1 ranged from 90 to 120% in muscle and 103 to 123% in liver. The range of variation for FB2 was 83 to 115% in muscle and 102 to 128% in liver (Table 2). The mean recovery rates of FB3 calculated as explained above, ranged from 91 to 121% in muscle and from 78 to 120% in liver. The limit of quantitation in muscle and liver was defined as 0.25 µg/kg for FB1, FB2, and FB3. This limit corresponds to the lowest concentration used in the spiked samples [41]. At this concentration, the lowest signal to noise ratios measured for FB1, FB2, and FB3 each were greater than 10, 3, and 5, respectively. The previous LOQ reported in muscle and liver for FB1 and FB2 with LC-MS/MS detection—but with no IA cleanup of samples—was 10 µg/kg, whereas the previous LOQ reported in liver for FB1 with IA cleanup and IF detection was 13 µg/kg [28,33,42]. 

The intra-day repeatability and inter-day reproducibility of the whole method were calculated on spiked samples and expressed by the relative standard deviation (RSD) of concentrations of C13FB1 and C13FB2. In muscles, the RSD of C13FB1 and C13FB2 ranged from 10 to 19% and 15 to 28%, respectively. The range of variation of the RSD in liver was 8 to 19% and 16 to 27%, respectively. These values in tissues are near the RSD of 14 and 28% respectively observed with FB1 and FB2 when solutions of standards were passed through IA column (Table 2). The RSD observed with this method were generally higher than those reported with methods that did not used IA cleanup of samples, but not significantly different from what was previously reported when IA cleanup of the liver was used before IF detection of FB1 [14,33,42]. It should be noted that whereas it has been reported that IA cleanup of sample enabled further detection of HFB1, this was not the case in the present study [43]. This difference is attributed to the antibodies used.

### 2.3. Measure of FB in Muscle and Liver of Animals Fed a Diet Containing FB

Figure 2C shows a typical chromatogram of a muscle from a turkey fed a diet containing 16.2, 3.98, and 5.18 mg/kg FB1, FB2, and FB3, respectively, and spiked with 2.5µg/kg of C13FB1 and C13FB2 each, for 14 days. Chromatograms of the liver resembled those of muscle. The concentrations in FB were measured after a starvation period of eight hours. The results observed in tissues of chickens and turkeys fed different diets containing fusariotoxins are shown in Table 3 and Table 4. Eight muscle and eight liver samples of each group were analyzed for FB levels in tissues. The concentrations of FB in the control diets were very low, less than 0.05 mg FB1+FB2. The concentration of FB in the liver of animals fed the control diets was less than 0.25 µg/kg, the LOQ, in all the samples analyzed except two, in which very low levels of FB (less than 1 µg/kg) were quantified. Two highly contaminated muscle samples containing more than 10 µg FB1/kg were found, one in chicken and the other in turkey. These two samples can be excluded from the group by a statistical test used for the exclusion of variable (Dixon, *p* < 0.001). As no FB was found in the liver of these two animals it was supposed that contamination occurred after sampling. Another sample of chicken muscle contained less than 1 µg/kg of FB. All the other samples contained less than 0.25 µg/kg (LOQ).

In chicken and turkey fed with the FB diet, the mean FB1 levels in livers were 44.7 and 41.5 µg FB1/kg, respectively. The small difference in the concentration of FB1 between chickens and turkey agrees with the slight difference in the quantities of FB in the feed. The concentration of FB1 in liver agrees with the results of previous studies conducted in turkey with a similar level of exposure [44]. The slight difference between chickens and turkeys suggests that the duration of exposure, which was 14 days in turkey and 35 in chickens, has only minor consequences for the concentration of FB in the liver. This observation is in agreement with the rapid half-life of elimination reported for FB1 suggesting that FBs have only weak ability to cumulate in tissues [10,14]. The mean concentration in FB1 in chicken and turkey muscle was 39 and 14% of the concentration found in liver, respectively. In chicken, the maximum FB1 concentration found in muscle samples was higher than the minimum concentration found in the liver samples, which was not the case in turkey. Because this study is the first one reporting FB1 in chicken and turkey muscles it is difficult to interpret these differences between species. The levels of FB2 and FB3 in tissues were always lower than those of FB1, in agreement with their respective exposure. The concentration of FB2 in liver and muscle was around three-fold higher than the concentration of FB3, both in chicken and turkey. This result is surprising because the FB2 levels measured in the diets were lower than those of FB3 in both experiments.

The consequences of the distribution of a diet containing FB as well as DON and ZEN on the levels of FB in tissues are shown in Table 3 and Table 4. Although levels of FB in liver and muscle generally resembled the levels reported when animals were fed with the diet containing FB alone, some differences can be highlighted. Because the FB and the FBDONZEN diets did not contain exactly the same amounts of FB, comparison can be done by calculating the tissue to feed ratio. In chickens fed the FB and the FBDONZEN diets, the FB1 liver to feed ratios were 0.21 and 0.37%, respectively, whereas the muscle to feed ratios were 0.83 and 1.15%, respectively (Table 3). In turkeys fed the FB and the FBDONZEN diets, the FB1 liver to feed ratios were 0.21 and 0.25%, respectively, whereas the muscle to feed ratio were 0.36 and 0.64%, respectively (Table 4). The consequences of feeding the FBDONZEN diet on the levels of FB2 and FB3 in liver and muscle were generally of the same nature as those observed with FB1. Taken together, these results suggest that the FBDONZEN diet slightly increased the concentration of FB in tissues, compared when the animals were fed the diet containing FB alone. These results agree with previous data suggesting weak interferences between fusariotoxins and oral bioavailability of drugs and nutrients. In a previous study it was shown that three weeks of exposure to 3.12 mg DON/kg feed had no influence on the oral bioavailability of FB1 administered as a single oral bolus of 2.5 mg FB/kg BW to broiler chickens [22]. Also, even though it has been found that feeding DON can promote the absorption of doxycycline and paromomycin in pig, the authors reported complex interactions between mycotoxins, mycotoxin binders, and antibiotics [16,45]. In another study conducted with ZEN and FB, it was reported that feeding 1 mg/kg of ZEN for 15 days slightly increased apparent digestibility of nutrients, but no interaction between ZEN and FB was found [24]. Thus, all the studies conducted to date demonstrated that feeding multiple fusariotoxins only has minor effects on the oral bioavailability of FB and its concentration in tissue, in agreement with the lack of interaction with health [34,35].

In conclusion, this study demonstrates for the first time the presence of FB in muscle of chickens and turkey fed at the highest recommended levels in FB in the EU in poultry feed. Mean cumulated concentrations of FB1, FB2, and FB3 in muscle and liver were around 20 and 50 µg/kg, respectively, while the highest cumulative concentrations found in some samples were above 60 and 100 µg/kg, respectively. Although further studies are necessary to investigate the fine effects of exposure to multiple fusariotoxins on the level of FB in tissues, these effects seem to be moderate.

## 3. Material and Methods

### 3.1. Tissue Samples

Tissue samples were obtained from chickens and turkeys fed with experimental diets to investigate the effects of fusariotoxins on health [34,35]. The experimental protocols were approved by the French Ministry of Higher Education and Research and registered under number 02032.01. Briefly, ground cultured toxigenic *Fusarium* strains were incorporated in corn–soybean diets formulated to best meet the nutritional needs of the animals. The control diets (Control) were free of mycotoxins, the fumonisin (FB) diets were formulated to contain 20 mg FB1+FB2/kg, and the fusariotoxin diets (FBDONZEN) were formulated to contain 20, 5, and 0.5 mg/kg of FB1+FB2, DON and ZEN, respectively. Each of the experimental diet was distributed ad libitum to 14 broilers from the 1st to the 35th day of age and to 14 turkeys from the 55th to the 70th day of age. After a starvation period of 8 h, the animals were killed by exsanguination after stunning by electrocution. The liver and the breast muscles were collected and stored at −80 °C until analysis. No signs of toxicity were observed, and only slight differences were found between groups on performance, organ weight, histopathology, intestinal morphometry and the number of goblet cells, oxidative damage, sphingolipid metabolism, and male reproductive toxicity [34,35].

### 3.2. Fumonisins, Reagents and LC-MS/MS Conditions

All reactive and reagents were purchased from Sharlab S.L. (Sentmenat, Spain). Standard solutions of FB1, FB2, FB3, U-[^13^C_34_]-FB1, and U-[^13^C_34_]-FB2 with certified concentrations of each analyte were purchased from Biopure (Tulln, Austria). The UPLC MS/MS system, including the software used to treat the chromatograms, was purchased from Agilent (Santa Clara, CA, USA). The UPLC system was a 1260 model composed of an automatic injector, a degasser, and a binary pump. A Poroshell 120 column (3.0 × 50 mm, 2.7 µm) was used for the separation step. A 6410 triple quad was used for detection after positive electrospray ionization. Source parameters were adjusted as follows: the temperature of the gas was set at 300 °C, gas flow at 10 L/minute, nebulizer was 25 psi, capillary voltage was 4000 V. Table 1 lists the optimized MRM conditions used for LC-ESI-MS/MS analysis for each analyte. The most abundant transition was chosen for MRM quantitation, while two other transitions were used as qualifiers for FB1, FB2, and FB3. Only one transition was used for qualification of C13FB1 and C13FB2.

The mobile phase was composed of a mixture of methanol (solvent A) and water (solvent B), each containing 0.1% formic acid (*v*/*v*), and was delivered at a flowrate of 0.3 mL/min. Solvents A and B were in the same proportion at the beginning of the run then a gradient of elution was introduced to reach 95% of A and 5% of B at 5 min. Return to the initial conditions was achieved at 8 min, then 4 min of washing was done before a new run was performed. The volume of injection was 10 microliters.

### 3.3. Analysis of Standards Solutions and Efficiency of Immunoaffinity Columns

Certified standards solutions were diluted in acetonitrile/water (1:1) to obtain working solutions containing mixture of FB1, FB2, and FB3 at 500 (FB500), 100 (FB100), and 20 ng/mL (FB20) and mixtures of C13FB1 and C13FB2 at 500 (C13FB500) and 100 ng/mL (C13FB100). Variables volumes of working solutions were evaporated to dryness. Dry residue was solubilized in 200 µL of mobile phase composed by a 50/50 mixture (*v*/*v*) of solvent A and B. Concentrations of each analyte in the injected solutions were 0, 2, 10, 50, and 100 ng/mL A quadratic fit of measured signal (y-axis) vs concentration (x-axis) was used. Accuracy was calculated at each concentration and was considered as acceptable for a relative standard deviation (RSD) of 20%.

The recovery rates of standards solutions passed through the IA columns were measured at different concentrations. Variable volumes of working solutions were solubilized in a final volume of 10 mL of ACN/MeOH/PBS (5:5:90, *v*/*v*/*v*) and passed through a FUMONIPREP column (R. Biopharm Rhone Ltd., Glasgow, Scotland) according to the manufacturer instructions. Columns were washed by 10 mL of 2mM pH 7.3 saline phosphate buffer (PBS), and eluted with 1.5 mL of methanol followed by 1.5 mL of water. The eluate was collected, evaporated to dryness, and stored at −20 °C until analysis. Before analysis, the dry residue was suspended in 200 µL of mobile phase composed by a mixture of solvent A and B (50:50, *v*/*v*) and sonicated for 5 min. Solubilized residue was centrifuged 10 min at 3000× *g*, the supernatant was collected and placed in chromatographic vials. Expected concentrations of FB1, FB2, and FB3 were 0, 2, 10, 50, and 100 ng/mL while expected concentrations of C13FB1 and C13FB2 were 62.5 ng/mL

### 3.4. Treatment of Tissue Samples and Determination of the Recovery Rates

Five g of muscle were homogenized in 5 mL of distilled water with an Ultra Turrax. Then 25 mg of NaCl, 25 µL of a working solution C13FB500, and 5 mL of acetonitrile/methanol (1:1) were added. Livers were prepared in the same conditions except 1 g of tissue was homogenized in 2mL of water and 2 mL of acetonitrile/methanol was added. Homogenized samples were placed on a stir table at 300 rpm for 2 h and centrifuged for 15 min at 3000× *g*. The supernatant was collected, 8 mL of hexane was added, and the mixture was vortexed for 30 seconds. The organic phase (upper) and the aqueous phase were separated by 15 min of centrifugation at 3000× *g*. For muscles samples, 5 mL of the aqueous phase were collected and 20 mL of 2mM pH 7.3 PBS were added. For the liver samples, 2 mL of the aqueous phase were collected and 8 mL of PBS were added. Extracts solubilized in PBS were passed through a FUMONIPREP column as previously described.

Matrix interactions were measured on tissue samples obtained from animals not exposed to FB in their diet over a period of at least 15 days. Muscle and liver were extracted and purified as previously described except the lack of fortification with C13FB500. Variable volumes of working solutions were added to the dry residue and evaporated to dryness. The dry residue was solubilized in 200 µL of mobile phase as previously described. Expected concentrations of FB1, FB2, and FB3 were 0, 2, 10, 50, and 100 ng/mL while expected concentrations of C13FB1 and C13FB2 were 62.5 ng/mL.

The recovery rates of standards solutions of FB were measured in fortified blank muscle and blank liver samples obtained from birds fed the mycotoxin-free diets. Tissue samples were prepared as previously described, fortified with 25 µL of IS500 and variable volumes of FB500, FB100, and FB20 to obtain supplementation levels equivalent to 0, 0.25, 1, and 5 ng/g of FB1, FB2, and FB3. Because FB1, FB2, and FB3 concentrations in diets are different, two other assays were performed to obtain final FB1, FB2, and FB3 concentrations of 25, 5, and 5 ng/g, and 100, 5, and 5 ng/g, respectively. In all the assays, the concentrations of C13FB1 and C13FB2 were 2.5ng/g in muscles and 12.5 ng/g in liver. The intra-day repeatability (n = 5) and inter-day reproducibility (5 days) of the whole method were calculated for C13FB1 and C13FB2 on muscle and liver spiked samples and expressed by the RSD of the concentrations measured. The LOQ was defined as the lowest concentration of a sample that can still be quantified with acceptable precision and accuracy (bias). The acceptance criteria for these two parameters were 20% RSD for precision and ± 20% for bias [41].

### 3.5. Statistical Analysis

The calibration curves obtained after passage of standard solutions on IA column and the calibration curves done to assess matrix effect in each species were compared to standard calibration curves using two-tailed paired t-test. Recovery rates, matrix effects and species effects were compared using ANOVA. When a significant difference was found (*p* < 0.05) a complementary comparison of mean was done using the Kruskall-Wallys test. Groups that are statistically different (*p* < 0.05) are identified by different letters.

## Figures and Tables

**Table 1:** MRM transitions and MS/MS parameters used for the fumonisins (FBs) analysis.

	FB1	FB2	FB3	C13FB1	C13FB2
Precursor * (M+1)	722.4	706	706	756.4	740
Quantifier *	334.4	336.4	336.4	356.5	358.6
Fragmentation/Collision (V)	190/41	190/37	190/37	190/40	160/36
Qualifier 1 * (Abundance, %)	352.4(94)	318.4(56)	318.4(44)	738.6(40)	340.5(97)
Fragmentation/Collision (V)	190/37	190/41	190/29	190/28	160/40
Qualifier 2 *(Abundance, %)	704.4(41)	354.5(22)	354.5(30)	-	-
Fragmentation/Collision (V)	190/29	190/33	190/25	-	-
Retention time (min)	6.02	6.95	6.58	6.01	6.97

* *m*/*z*

**Table 2:** FB recovery after immuno-affinity clean-up and matrix interactions observed in muscle and liver of chicken and turkey.

		FB1	FB2	FB3	C13FB1	C13FB2
Recovery after IA	(%)^1,2^	75 ± 9 ^A^	49 ± 13 ^B^	62 ± 13 ^A,B^	78 ± 11 ^A^	50 ± 14 ^B^
	slope	0.7943	0.4784	0.6279	-	-
Matrix interaction, muscle	(%) ^1,3^	104 ± 5 ^A^	59 ± 4 ^B^	92 ± 6 ^A^	98 ± 6 ^A^	58 ± 7
	slope	1.0722	0.6103	0.9658	-	-
Matrix interaction, liver	(%) ^1,3^	97 ± 16 ^A^	46 ± 6 ^B^	83 ± 7 ^A^	95 ± 14 ^A^	51 ± 5 ^B^
	slope ^3^	0.9203	0.4423	0.8088	-	-
Recovery muscle 0.25	(µg/kg) ^4^	0.28 ± 0.04	0.28 ± 0.03	0.29 ± 0.04	42 ± 4	17 ± 2
	%	112	115	110		
Recovery muscle 1	(µg/kg) ^4^	1.16 ± 0.04	1.13 ± 0.14	1.19 ± 0.09		
	%	116	113	119		
Recovery muscle 5	(µg/kg) ^4^	4.51 ± 0.36	5.07 ± 0.38	4.56 ± 0.39		
	%	90	101	91		
Recovery muscle 25/5/5	(µg/kg) ^4^	30.12 ± 4.47	4.13 ± 0.85	6.06 ± 0.99		
	%	120	83	121		
Recovery liver 0.25	(µg/kg) ^4^	0.26 ± 0.03	0.28 ± 0.03	0.28 ± 0.03	75 ± 8	37 ± 4
	%	103	111	110		
Recovery liver 1	(µg/kg) ^4^	1.12 ± 0.19	1.05 ± 0.16	1.20 ± 0.22		
	%	112	102	120		
Recovery liver 5	(µg/kg) ^4^	5.54 ± 0.19	5.77 ± 0.86	5.68 ± 0.38		
	%	111	115	114		
Recovery liver 25/5/5	(µg/kg) ^4^	28.81 ± 4.1	6.42 ± 0.4	4.24 ± 0.71		
	%	115	128	85		
Recovery liver 100/5/5	(µg/kg) ^4^	122.96 ± 6.37	6.29 ± 0.68	3.92 ± 0.77		
	%	123	126	78		

^1^ Mean ± SD of three determinations performed at 2, 10, 50, and 100 ng/mL for FB1, FB2, and FB3 and 62.5 ng/mL for C13FB1 and C13FB2. When a significant difference was found (ANOVA, *p* < 0.05) a complementary comparison of mean was done using the Kruskall-Wallys test. Groups that are statistically different (*p* < 0.05) are identified by different letters. ^2^ Solutions of standard diluted in MetOH/H_2_O. ^3^ Blank extracts purified on IA column obtained in muscles (5g) and livers (1g) of chicken (n = 4) and turkeys (n = 4) not exposed to mycotoxins in feed over at least 14 days. Effect of animal species was not significant (*p* > 0.05). ^4^ Mean ± SD measured in blank muscles (5g) and blank livers (1g) samples spiked at different concentration of FB1, FB2, and FB3 and C13FB1 + C13FB2 each at 12.5 µg/kg in muscle and 62.5 µg/kg in liver. Concentrations of FB1 and FB2 in samples were calculated by taking into account the recovery rate measured on each sample for C13FB1 and C13FB2, respectively. Concentration of FB3 was calculated by taking into account the recovery rate of C13FB1 corrected by the ratio of the slope measured on standards solution for FB1 and FB3 after IA cleanup.

**Table 3:** FB concentrations in muscle and liver of chicken fed lifetime with mycotoxins contaminated diets.

		FB1	FB2	FB3
Duration of exposure (days)		35	35	35
Feed Control (µg/kg)	1 to 10 d	35	<10	<10
	11 to 35 d	25	<10	<10
Liver	positive/total	1/8	1/8	1/8
	max; min (µg/kg)	0.59; <0.25	0.33; <0.25	0.25; <0.25
Muscle	positive/total	2/8	2/8	2/8
	max; min (µg/kg)	13.59; <0.25	2.4; <0.25	0.95; <0.25
Feed FB (µg/kg)	1 to 10 d	19,500	1600	2000
	11 to 33 d	21,000	2130	2300
Liver (µg/kg)	mean ± SD	44.7 ± 20.61	2.61 ± 1.39	0.79 ± 0.31
	max; min	85.64; 27.99	5.4; 1.32	1.17; 0.36
Muscle (µg/kg)	mean ± SD	17.5 ± 16.84	3.39 ± 2.58	1.26 ± 1.02
	max; min	48.13; 3	7.68; 0.58	3.05; 0.35
Feed FBDONZEN (µg/kg) ^1^	1 to 10 d	17,600	1440	2050
	11 to 35 d	17,700	1530	2030
Liver (µg/kg)	mean ± SD	65.98 ± 13.12	4.19 ± 1.45	1.63 ± 0.39
	max; min	87.05; 50.82	6.87; 2.8	2.19; 1.32
Muscle (µg/kg)	mean ± SD	20.39 ± 21.17	3.3 ± 4.02	1.41 ± 1.71
	max; min	50.56; 4.62	9.32; 0.92	3.95; 0.34

^1^ Concentrations of DON and ZEN were 3820 and 415 µg/kg in the diet fed from the 1st to the 10th day of age and 4170 and 430 µg/kg in the diet fed from the 11th to the 35th day of age, respectively.

**Table 4:** FB concentrations in muscle and liver of turkeys fed with mycotoxins contaminated diets from 55 to 70 days of age.

		FB1	FB2	FB3
Duration of exposure (days)		14	14	14
Feed Control (µg/kg)		20	<10	<10
Liver	positive/total	1/8	0/8	1/8
	max; min (µg/kg)	0.60; <0.25	<0.25	0.32<0.25
Muscle	positive/total	1/8	1/8	1/8
	max; min (µg/kg)	19.74; <0.25	4.89; <0.25	1.52; <0.25
Feed FB (µg/kg)		16,200	3980	5,180
Liver (µg/kg)	mean ± SD	41.47 ± 13.57	4.23 ± 2.78	1.41 ± 0.69
	max; min	63.09; 27.61	10.03; 0.91	2.77; 0.65
Muscle (µg/kg)	mean ± SD	5.77 ± 3.79	1.52 ± 0.69	0.54 ± 0.32
	max; min	10.01; 1	2.28; 0.51	0.88; <0.25
Feed FBDONZEN (µg/kg) ^1^		21,500	4200	6010
Liver (µg/kg)	mean ± SD	53.8 ± 20.14	4.11± 2.89	1.86 ± 0.99
	max; min	94.25; 35.74	9.75; 2	3.79; 0.92
Muscle (µg/kg)	mean ± SD	13.94 ± 14	3.02 ± 3.49	1.22 ± 1.29
	max; min	32.82; 2.19	8.01; 0.51	2.96; <0.25

^1^ Concentrations of DON and ZEN were 5150 and 570 µg/kg, respectively.

## Abbreviations

FB	Fumonisins B
FUS	Fusariotoxins
FB_1_	Fumonisin B_1_
FB_2_	Fumonisin B_2_
FB_3_	Fumonisin B_3_
FA	Fumonisin A
FP	Fumonisin P
HFB	Hydrolyzed Fumonisins
TCA	Tricarballylic acid
DON	Deoxynivalenol
ZEN	Zearalenone
IA	Immunoaffinity
RSD	Relative Standard Deviation
BW	Body Weight
